# Risk factors associated with monozygotic twinning in offspring conceived by assisted reproductive technology

**DOI:** 10.1093/hropen/hoad035

**Published:** 2023-09-14

**Authors:** Na Chen, Jingyu Li, Yexing Li, Yiyuan Zhang, Jiarong Li, Jie Gao, Jingmei Hu, Linlin Cui, Zi-Jiang Chen

**Affiliations:** Center for Reproductive Medicine, Cheeloo College of Medicine, Shandong University, Jinan, Shandong, China; Research Unit of Gametogenesis and Health of ART-Offspring, Chinese Academy of Medical Sciences (No.2021RU001), Jinan, Shandong, China; Key Laboratory of Reproductive Endocrinology of Ministry of Education, Shandong University, Jinan, Shandong, China; Shandong Key Laboratory of Reproductive Medicine, Jinan, Shandong, China; Shandong Provincial Clinical Research Center for Reproductive Health, Jinan, Shandong, China; Shandong Technology Innovation Center for Reproductive Health, Jinan, Shandong, China; National Research Center for Assisted Reproductive Technology and Reproductive Genetics, Shandong University, Jinan, Shandong, China; Center for Reproductive Medicine, Cheeloo College of Medicine, Shandong University, Jinan, Shandong, China; Research Unit of Gametogenesis and Health of ART-Offspring, Chinese Academy of Medical Sciences (No.2021RU001), Jinan, Shandong, China; Key Laboratory of Reproductive Endocrinology of Ministry of Education, Shandong University, Jinan, Shandong, China; Shandong Key Laboratory of Reproductive Medicine, Jinan, Shandong, China; Shandong Provincial Clinical Research Center for Reproductive Health, Jinan, Shandong, China; Shandong Technology Innovation Center for Reproductive Health, Jinan, Shandong, China; National Research Center for Assisted Reproductive Technology and Reproductive Genetics, Shandong University, Jinan, Shandong, China; Center for Reproductive Medicine, Cheeloo College of Medicine, Shandong University, Jinan, Shandong, China; Research Unit of Gametogenesis and Health of ART-Offspring, Chinese Academy of Medical Sciences (No.2021RU001), Jinan, Shandong, China; Key Laboratory of Reproductive Endocrinology of Ministry of Education, Shandong University, Jinan, Shandong, China; Shandong Key Laboratory of Reproductive Medicine, Jinan, Shandong, China; Shandong Provincial Clinical Research Center for Reproductive Health, Jinan, Shandong, China; Shandong Technology Innovation Center for Reproductive Health, Jinan, Shandong, China; National Research Center for Assisted Reproductive Technology and Reproductive Genetics, Shandong University, Jinan, Shandong, China; Center for Reproductive Medicine, Cheeloo College of Medicine, Shandong University, Jinan, Shandong, China; Research Unit of Gametogenesis and Health of ART-Offspring, Chinese Academy of Medical Sciences (No.2021RU001), Jinan, Shandong, China; Key Laboratory of Reproductive Endocrinology of Ministry of Education, Shandong University, Jinan, Shandong, China; Shandong Key Laboratory of Reproductive Medicine, Jinan, Shandong, China; Shandong Provincial Clinical Research Center for Reproductive Health, Jinan, Shandong, China; Shandong Technology Innovation Center for Reproductive Health, Jinan, Shandong, China; National Research Center for Assisted Reproductive Technology and Reproductive Genetics, Shandong University, Jinan, Shandong, China; Center for Reproductive Medicine, Cheeloo College of Medicine, Shandong University, Jinan, Shandong, China; Research Unit of Gametogenesis and Health of ART-Offspring, Chinese Academy of Medical Sciences (No.2021RU001), Jinan, Shandong, China; Key Laboratory of Reproductive Endocrinology of Ministry of Education, Shandong University, Jinan, Shandong, China; Shandong Key Laboratory of Reproductive Medicine, Jinan, Shandong, China; Shandong Provincial Clinical Research Center for Reproductive Health, Jinan, Shandong, China; Shandong Technology Innovation Center for Reproductive Health, Jinan, Shandong, China; National Research Center for Assisted Reproductive Technology and Reproductive Genetics, Shandong University, Jinan, Shandong, China; Center for Reproductive Medicine, Cheeloo College of Medicine, Shandong University, Jinan, Shandong, China; Research Unit of Gametogenesis and Health of ART-Offspring, Chinese Academy of Medical Sciences (No.2021RU001), Jinan, Shandong, China; Key Laboratory of Reproductive Endocrinology of Ministry of Education, Shandong University, Jinan, Shandong, China; Shandong Key Laboratory of Reproductive Medicine, Jinan, Shandong, China; Shandong Provincial Clinical Research Center for Reproductive Health, Jinan, Shandong, China; Shandong Technology Innovation Center for Reproductive Health, Jinan, Shandong, China; National Research Center for Assisted Reproductive Technology and Reproductive Genetics, Shandong University, Jinan, Shandong, China; Center for Reproductive Medicine, Cheeloo College of Medicine, Shandong University, Jinan, Shandong, China; Research Unit of Gametogenesis and Health of ART-Offspring, Chinese Academy of Medical Sciences (No.2021RU001), Jinan, Shandong, China; Key Laboratory of Reproductive Endocrinology of Ministry of Education, Shandong University, Jinan, Shandong, China; Shandong Key Laboratory of Reproductive Medicine, Jinan, Shandong, China; Shandong Provincial Clinical Research Center for Reproductive Health, Jinan, Shandong, China; Shandong Technology Innovation Center for Reproductive Health, Jinan, Shandong, China; National Research Center for Assisted Reproductive Technology and Reproductive Genetics, Shandong University, Jinan, Shandong, China; Center for Reproductive Medicine, Cheeloo College of Medicine, Shandong University, Jinan, Shandong, China; Research Unit of Gametogenesis and Health of ART-Offspring, Chinese Academy of Medical Sciences (No.2021RU001), Jinan, Shandong, China; Key Laboratory of Reproductive Endocrinology of Ministry of Education, Shandong University, Jinan, Shandong, China; Shandong Key Laboratory of Reproductive Medicine, Jinan, Shandong, China; Shandong Provincial Clinical Research Center for Reproductive Health, Jinan, Shandong, China; Shandong Technology Innovation Center for Reproductive Health, Jinan, Shandong, China; National Research Center for Assisted Reproductive Technology and Reproductive Genetics, Shandong University, Jinan, Shandong, China; Center for Reproductive Medicine, The Second Hospital, Shandong University, Jinan, Shandong, China; Center for Reproductive Medicine, Cheeloo College of Medicine, Shandong University, Jinan, Shandong, China; Research Unit of Gametogenesis and Health of ART-Offspring, Chinese Academy of Medical Sciences (No.2021RU001), Jinan, Shandong, China; Key Laboratory of Reproductive Endocrinology of Ministry of Education, Shandong University, Jinan, Shandong, China; Shandong Key Laboratory of Reproductive Medicine, Jinan, Shandong, China; Shandong Provincial Clinical Research Center for Reproductive Health, Jinan, Shandong, China; Shandong Technology Innovation Center for Reproductive Health, Jinan, Shandong, China; National Research Center for Assisted Reproductive Technology and Reproductive Genetics, Shandong University, Jinan, Shandong, China; Shanghai Key Laboratory for Assisted Reproduction and Reproductive Genetics, Shanghai, China; Center for Reproductive Medicine, Ren Ji Hospital, School of Medicine, Shanghai Jiao Tong University, Shanghai, China

**Keywords:** ART, single embryo transfer (SET), frozen embryo transfer (FET), monozygotic (MZ) twins, blastocyst, characteristics of infertile populations, gamete or embryo manipulations, classification of blastocyst, IVF, ICSI

## Abstract

**STUDY QUESTION:**

What are the factors influencing the occurrence of monozygotic (MZ) twins in offspring conceived by assisted reproductive technology (ART)?

**SUMMARY ANSWER:**

Parental ages, the transfer of fresh versus frozen embryos, and the grade of blastocysts are all related to MZ twinning in ART offspring.

**WHAT IS KNOWN ALREADY:**

Offspring conceived by ART have significantly increased risk of MZ twins, which may be due to the characteristics of the infertile population. The objective of this study was to explore the incidence of monozygotic (MZ) twins after ART and to clarify the risk factors for MZ twinning.

**STUDY DESIGN, SIZE, DURATION:**

A total of 255 monozygotic twins were enrolled in this cohort study, and then matched with singletons at a ratio of 1:4 randomly (with 1020 in the control group). All offspring were conceived by single embryo transfer.

**PARTICIPANTS/MATERIALS, SETTING, METHODS:**

The collected data were divided into the following three aspects for analysis: characteristics of the infertile population, gamete or embryo manipulations, and factors related to embryo development.

**MAIN RESULTS AND THE ROLE OF CHANCE:**

The incidence of MZ twins was 1.638% (255 out of 15 567 pregnancies after single embryo transfers). Compared to singleton births, a significantly lower rate of frozen embryo transfers (FET; 78.0% vs 86.1% *P* = 0.002) was seen amongst the MZ twins. Amongst fresh ETs, the rate of blastocyst transfers in the MZ twins group was higher compared to that in the control group (92.9% vs 75.4%, *P* = 0.005). We also found that certain grades of blastocysts in terms of trophectoderm (TE) development, inner cell mass + TE development and the classification of ‘top-quality’ embryos were associated with the incidence of MZ twinning (*P* = 0.025, *P* = 0.012, *P* = 0.020, respectively). Logistic regression analysis revealed that higher paternal age (odds ratio (OR) = 0.94, 95% CI = 0.89–1.00, *P* = 0.029) and FET (OR = 0.48, 95% CI = 0.33–0.68, *P* = 0.001) may be protective factors against MZ twinning. However, higher maternal age (OR = 1.07, 95% CI = 1.01–1.13, *P* = 0.027) and the transfer of blastocysts (OR = 4.31, 95% CI = 1.46–12.73, *P* = 0.008) appeared to be associated with an increased risk of MZ twinning. Amongst blastocyst transfers, a C grade TE may be protective factor against MZ twinning (B: OR = 1.90, 95% CI = 1.18–3.07, *P* = 0.009; A: OR = 1.58, 95% CI = 0.93–2.67, *P* = 0.089).

**LIMITATIONS, REASONS FOR CAUTION:**

First, our definition of MZ twins was based on twins’ birth after single embryo transfers (SET), rather than ultrasound examination during early pregnancy. Second, the parental characteristics of the two groups were homogenous, so it was difficult to find any associations between infertility factors and the incidence of MZ twins.

**WIDER IMPLICATIONS OF THE FINDINGS:**

This multifaceted analysis of the risk factors for MZ twinning provides some information for clinical interventions in high-risk populations.

**STUDY FUNDING/COMPETING INTEREST(S):**

This study was supported by the National Key Technology Research and Developmental Program of China (2022YFC2704404), CAMS Innovation Fund for Medical Sciences (2021-I2M-5-001), Taishan Scholars Program for Young Experts of Shandong Province (tsqn201909195), the Basic Science Center Program (31988101), and the Shandong Provincial Key Research and Development Program (2020ZLYS02). All authors have no conflicts of interest to declare.

**TRIAL REGISTRATION NUMBER:**

N/A.

WHAT DOES THIS MEAN FOR PATIENTS? With the widespread use of assisted reproductive technology (ART), over 10 million ART offspring have been born. Some studies have pointed out that the incidence of identical (monozygotic) twins is significantly increased in ART offspring, which may be related to the infertility characteristics of their parents, although gamete manipulation may also play a role, and the influencing factors have still not been determined. This study analysed the differences in parental characteristics and embryonic characteristics between singletons and monozygotic twins. The results showed that advanced maternal age, fresh embryo transfers, blastocyst transfers and high-embryo gradings may all be associated with monozygotic twinning in ART pregnancies.

## Introduction

Monozygotic (MZ) twins are offspring originating from a single zygote that divides and grows into two independent embryos. The incidence rate of MZ twins following natural conception is about 1 in 250 births (Hankins and Saade, 2005). However, with the development of ART, the number of MZ twins has significantly increased. Parazzini *et al.* (2016) reported that the incidence rate of MZ twins was 60% higher after ART. Although the incidence of MZ twinning has varied greatly among studies, the overall trend was that the chance of MZ twins was higher with assisted reproduction than with natural conceptions.

Twin pregnancies have been shown to be associated with increased risks of maternal, obstetric and perinatal complications (Duffy, 2021). The risk of adverse outcomes in twins was reported to be four times higher than that in singleton births (Evans *et al.*, 2022). Complications, such as twin-to-twin transfusion syndrome (TTTS) and selective intrauterine growth restriction, have been reported with MZ twins. TTTS occurs in 10–15% of monochorionic pregnancies, which may lead to a higher risk of perinatal mortality (Bhat, 2010). Thus, considering the available evidence on twin pregnancies, MZ twinning in particular can have an adverse effect on maternal and foetal health.

Studies have explored the factors that influence the rate of MZ twins. Maternal age was considered a factor affecting the development of MZ twins in many studies; however, the findings have been inconclusive and inconsistent. Some studies have reported higher incidence rates of MZ twins in younger women because of healthy ovarian function (Knopman *et al.*, 2014; Sobek *et al.*, 2015). In contrast, advanced maternal age may be associated with a thinner zona pellucida and an increase the risk of MZ twins (Knopman *et al.*, 2010). Furthermore, genetic factors, maternal body mass index (BMI), the cause of infertility and embryo morphology are also thought to be related to the incidence of MZ twins (Hviid *et al.*, 2018).

The mechanisms of MZ twinning and the adverse effects of these twins on maternal and infant health remain unclear. The purpose of this study was to investigate the factors that influence incidence of MZ twins after ART for a better understanding of the mechanisms.

## Materials and methods

### Participants

In this retrospective case–control study, all participants were recruited from the Center for Reproductive Medicine, Shandong University; all had received elective singleton embryo transfer between 2007 and 2019. A total of 15 567 pregnancies were achieved. Three cases of triplet births (0.019%), 15 256 cases of singleton births (98.002%) and 308 cases of twin births (1.978%) were reported. Cases of donated sperm and oocytes (N = 1067), opposite-sex twins (N = 26), cases of triplets or higher multiples (N = 3), and multifetal pregnancy reduction cases and stillbirths (N = 9) were excluded. Overall, 255 (1.638%) twins were included in the MZ twins group and 1020 singletons were randomly matched in a 1:4 ratio to form the control group. Appropriate institutional review boards approved the study. All mothers provided written informed consent.

### Measurements

The collected data were divided as follows for analysis: (i) characteristics of the infertile population, i.e. parental age, BMI and aetiology of infertility; (ii) gamete or embryo manipulations in ART, i.e. protocols of ovarian stimulation, the total dose of hCG used during ovarian stimulation, IVF versus ICSI, preimplantation genetic testing (PGT), fresh embryo transfer (ET) versus frozen ET (FET), and blastocyst versus cleavage embryo transfer; and (iii) embryonic factors, i.e. blastocyst grading. Blastocysts were graded according to three morphological features: blastocyst expansion and development of inner cell mass (ICM) and trophectoderm (TE) development (Gardner and Schoolcraft, 1999; Yin *et al.*, 2016). Development scores between 6 and 2 indicated a middle expanding blastocyst, an expanded blastocyst, a fully expanded blastocysts, an expanded blastocysts with partial hatching and a fully hatched blastocysts, respectively, while a score of 1 indicated an early blastocyst. Based on the number of cells and the degree of compactness in the arrangement, the ICM and TE were graded into A, B, and C (Schoolcraft *et al.*, 1999; Hong *et al.*, 2020). Based on the sample situation, ICM and TE were classified as AA, AB/BA, BB or AC/BC/CB. Alternatively, high-quality embryos were classified into grades 4AA, 4AB, 4BA, and 4BB, while all other lower-quality embryos were graded as lower than 4BB.

### Statistics

Continuous variables were expressed as mean ± SD, whereas categorical variables were expressed as percentages (numbers). All the continuous variables were tested for normality. Student’s *t*-test was used for analysing normally distributed variables, and the Mann–Whitney *U*-test was used for variables not following a normal distribution. Categorical variables were compared using the chi-squared test or Fisher’s exact test. Logistic regression analysis was performed to further evaluate the effect of factors influencing the incidence rate of MZ twins. The effects were described as odds ratio (OR) for logistic regressions with 95% CIs. A *P*-value <0.05 was considered statistically significant. All statistical analyses were performed using the Statistical Package for the Social Sciences (SPSS), Version 26.0 software.

## Results

The incidence of MZ twins after ART was 1.638% (255 out of 15 567), which was considerably higher than the reported incidence after spontaneous conception (∼0.4%) (Hankins and Saade, 2005).

We analysed the characteristics of the infertile population and did not find any significant differences between parents of singletons and MZ twins. After analysing ART manipulations (including the protocols of ovarian stimulation, doses of hCG, types of ART and types of transferred embryos), we found that the FET proportion was significantly lower in the MZ twins group than that in the singleton group (78.0% vs 86.1%, *P* = 0.002, Table 1). To avoid the influence of FET, we then performed stratified analysis as shown in Table 2. Among the fresh embryo transfers, the rate of blastocyst transfer in the MZ twins group was higher compared to that in the control group (92.9% vs 75.4%, *P* = 0.005).

**Table 1.** Population characteristics amongst monozygotic twin and singleton pregnancies.

	MZ twins	Singletons	*P* value
**N**	255	1020	
**Characteristics of infertile population**			
Maternal age (years), mean ± SD	30.6 ± 4.08	30.4 ± 4.19	0.263
<35 years, % (n)	84.3 (215)	85.2 (869)	0.724
≥35 years, % (n)	15.7 (40)	14.8 (151)	
Maternal BMI (kg/m^2^), mean ± SD	23.57 ± 3.50	23.34 ± 3.86	0.195
Fallopian tube diseases, % (n)	74.1 (189)	74.9 (764)	0.797
Endometriosis, % (n)	3.9 (10)	4.3 (44)	0.781
Ovulation disorders, % (n)	42.0 (107)	39.7 (405)	0.511
Paternal age (years), mean ± SD	31.09 ± 4.34	31.32 ± 4.92	0.988
Paternal BMI (kg/m^2^), mean ± SD	25.72 ± 4.00	25.81 ± 4.24	0.771
Oligoasthenospermia, asthenospermia, or azoospermia, % (n)	40.8 (104)	41.7 (425)	0.798
Teratospermia, % (n)	47.5 (121)	47.2 (481)	0.933
**Manipulations in ART**			
Ovarian stimulation			
Short protocol, % (n)	16.9 (43)	14.3 (146)	0.219
Long protocol, % (n)	53.7 (137)	56.6 (577)	
GnRH-ant protocol, % (n)	26.7 (68)	23.9 (244)	
Other protocols, % (n)	2.7 (7)	5.2 (53)	
Dose of gonadotrophins (IU), mean ± SD	1791.67 ± 842.56	1848.78 ± 917.93	0.420
Gamete or embryo manipulations			
IVF, % (n)	64.7 (165)	64.9 (662)	0.051
ICSI, % (n)	30.6 (78)	26.2 (267)	
PGT, % (n)	4.7 (12)	8.9 (91)	
Frozen embryo transfer, % (n)	**78.0 (199)**	**86.1 (878)**	**0.002**
Long culture duration, % (n)	98.4 (251)	96.5 (984)	0.108

Data are shown as mean ± SD or percentage (number) with statistically significant differences are expressed in bold (*P* < 0.05).

GnRH-ant: gonadotropin releasing hormone antagonist; MZ: monozygotic; BMI: body mass index; PGT: preimplantation genetic testing; SD, standard deviation.

**Table 2.** Stratified analysis of results regarding monozygotic twin and singleton pregnancies.

	Fresh embryo			Frozen embryo		
	MZ Twins	Singletons	*P*-value	MZ Twins	Singletons	*P*-value
**N**	56	142		199	878	
**Characteristics of infertile population**						
Maternal age (years), mean ± SD	31.42 ± 3.58	31.51 ± 4.39	0.921	30.41 ± 4.19	30.22 ± 4.13	0.477
<35 years, % (n)	82.1 (46)	76.1 (108)	0.354	84.9 (169)	86.7 (761)	0.516
≥35 years, % (n)	17.9 (10)	23.9 (34)		15.1 (30)	13.3 (117)	
Maternal BMI (kg/m^2^), mean ± SD	23.61 ± 3.14	23.98 ± 5.03	0.82	23.56 ± 3.61	23.24 ± 3.63	0.242
Fallopian tube diseases, % (n)	80.4 (45)	76.1 (108)	0.515	72.4 (144)	74.7 (656)	0.493
Endometriosis, % (n)	1.8 (1)	4.2 (6)	0.675	4.5 (9)	4.3 (38)	0.903
Ovulation disorders, % (n)	39.3 (22)	43.0 (61)	0.637	42.7 (85)	39.2 (344)	0.358
Paternal age (years), mean ± SD	32.04 ± 4.21	32.31 ± 5.14	0.857	30.83 ± 4.35	31.16 ± 4.87	0.707
<35 years, % (n)	78.6 (44)	72.5 (103)	0.382	82.9 (165)	80.4 (706)	0.417
≥35 years, % (n)	21.4 (12)	27.5 (39)		17.1 (34)	19.6 (172)	
Paternal BMI (kg/m^2^), mean ± SD	26.48 ± 4.36	26.02 ± 4.69	0.365	25.50 ± 3.88	25.78 ± 4.17	0.383
Oligoasthenospermia, asthenospermia, or azoospermia, % (n)	41.1 (23)	40.1 (57)	0.904	40.7 (81)	41.9 (368)	0.755
Teratospermia, % (n)	41.1 (23)	48.6 (69)	0.339	49.2 (98)	46.9 (412)	0.554
**Manipulations in ART**						
Ovarian stimulation						
Short protocol, % (n)	21.4 (12)	21.1 (30)	0.181	15.6 (31)	13.2 (116)	0.423
Long protocol, % (n)	46.4 (26)	56.3 (80)		55.8 (111)	56.6 (497)	
GnRH-ant protocol, % (n)	28.6 (16)	15.5 (22)		26.1 (52)	25.3 (222)	
Other protocols, % (n)	3.6 (2)	7.0 (10)		2.5 (5)	4.9 (43)	
Dose of gonadotrophins (IU), mean ± SD	1976 ± 984	1869 ± 919	0.527	1740 ± 793	184 ± 918	0.207
Gamete or embryo manipulations						
IVF, % (n)	67.9 (38)	75.4 (107)	0.487	63.8 (127)	63.2 (555)	0.097
ICSI, % (n)	30.4 (17)	23.2 (33)		30.7 (61)	26.7 (234)	
PGT, % (n)	1.8 (1)	1.4 (2)		5.5 (11)	10.1 (89)	
Blastocyst transfer, % (n)	**92.9 (52)**	**75.4 (107)**	**0.005**	100.0 (199)	99.9 (877)	1

Data are shown as mean ± SD or percentage (number). Indicators with statistically significant differences are expressed in bold (*P* < 0.05).

MZ: monozygotic; BMI: body mass index; GnRH-ant: gonadotropin releasing hormone antagonist; PGT: preimplantation genetic testing; SD, standard deviation.

We also analysed blastocyst grading in each group and found that the grading of TE and ICM+TE and the classification of top-quality embryos were associated with the incidence of MZ twins (*P* = 0.025, *P* = 0.012, *P* = 0.020, respectively). Embryos with a lower score were less represented in the MZ twins group compared to the singleton group (TE-C: 8.8% vs 14.7%; ICM + TE-AC/BC/CB: 8.8% vs 14.8%; quality of embryos lower than that of 4BB: 54.6% vs 62.3%) (Table 3).

**Table 3.** Comparison of blastocyst scores amongst monozygotic twin and singleton pregnancies.

	MZ twins	Singletons	*P* value
**N**	251	984	
Development score			
3, % (n)	0.8 (2)	0.8 (8)	0.302
4, % (n)	49.8 (125)	43.5 (428)	
5, % (n)	47.4 (119)	52.6 (518)	
6, % (n)	2.0 (5)	3.0 (30)	
ICM			
A, % (n)	27.1 (68)	31.2 (307)	0.207
Non A, % (n)	72.9 (183)	68.8 (677)	
TE			
A, % (n)	**25.1 (63)**	**26.7 (263)**	**0.025**
B, % (n)	**66.1 (166)**	**58.5 (576)**	
C, % (n)	**8.8 (22)**	**14.7 (145)**	
ICM + TE			
AA, % (n)	**16.3 (41)**	**20.8 (205)**	**0.012**
AB/BA, % (n)	**19.5 (49)**	**16.2 (159)**	
BB, % (n)	**55.4 (139)**	**48.2 (474)**	
BC/CB/AC, % (n)	**8.8 (22)**	**14.8 (146)**	
Top-quality embryos			
4AA, % (n)	**8.8 (22)**	**8.8 (87)**	**0.020**
4AB, % (n)	**5.2 (13)**	**5.0 (49)**	
4BA, % (n)	**4.8 (12)**	**1.7 (17)**	
4BB, % (n)	**26.7 (67)**	**22.2 (218)**	
Others,* % (n)	**54.6 (137)**	**62.3 (613)**	

Data are shown as percentage (number). Indicators with statistically significant differences are expressed in bold (*P* < 0.05).

* Embryos with quality lower than that of 4BB.

ICM: inner cell mass; TE: trophectoderm; MZ: monozygotic.

Regression analysis on the above-reported factors affecting MZ twins showed that paternal age (OR = 0.94, 95% CI = 0.89–1.00, *P* = 0.029) and FET (OR = 0.48, 95% CI = 0.33–0.68, *P* = 0.001) may be slightly protective against MZ twinning. In contrast, maternal age (OR = 1.07, 95% CI = 1.01–1.13, *P* = 0.027) and blastocyst transfer (OR = 4.31, 95% CI = 1.46–12.73, *P* = 0.008) appeared to be associated with an increased risk of MZ twins (Table 4).

**Table 4.** Logistic regression models of risk factors for MZ twinning in all offspring.

	OR (95% CI)	*P* value
Maternal age (years)	**1.07 (1.01–1.13)**	**0.027**
Maternal BMI	1.01 (0.98**–**1.05)	0.517
Paternal age (y)	**0.94 (0.89–1.00)**	**0.029**
Paternal BMI	0.99 (0.96–1.02)	0.561
Gamete or embryo manipulations		
IVF	Reference	
ICSI	1.22 (0.90–1.66)	0.209
PGT	0.59 (0.32–1.12)	0.106
Frozen embryo transfer	**0.48 (0.33–0.68)**	**0.001**
Blastocyst transfer	**4.31 (1.46–12.73)**	**0.008**

Indicators with statistically significant differences are expressed in bold (*P* < 0.05).

BMI: body mass index; PGT: preimplantation genetic testing; OR: odds ratio; CI: confidence interval.

Results of logistic regression analyses in blastocyst transfers (Table 5) showed that the C grade for TE may be relatively protective against MZ twinning compared to B grade blastocysts (OR = 1.90, 95% CI = 1.18–3.07, *P* = 0.009). Further, compared to the BC/CB/AC grade, the ICM+TE grade of BB or AB/BA may increase the chance of MZ twins (BB: OR = 1.95, 95% CI = 1.20–3.17, *P* = 0.007; AB/BA: OR = 2.05, 95% CI = 1.18–3.55, *P* = 0.011). A similar trend for the grade of AA was not observed possibly because of the sample size. Top-quality embryos of grade 4BA also increased the chance of MZ twins compared to poor-quality embryos (OR = 3.16, 95% CI = 1.47–6.77, *P* = 0.003). Overall, the results indicate that a C grade for TE may be protective against MZ twinning.

**Table 5.** Logistic regression models of blastocyst scores with regard to risk of MZ twinning.

	OR (95% CI)	*P* value
Development score		
3	Reference	
4	1.17 (0.25–5.57)	0.845
5	0.92 (0.19–4.38)	0.916
6	0.67 (0.11–4.10)	0.662
ICM		
Non A	Reference	
A	0.82 (0.60–1.12)	0.207
TE		
C	Reference	
B	**1.90 (1.18–3.07)**	**0.009**
A	1.58 (0.93–2.67)	0.089
ICM+TE		
BC/CB/AC (<4)	Reference	
BB (4)	**1.95 (1.20–3.17)**	**0.007**
AB/BA (6)	**2.05 (1.18–3.55)**	**0.011**
AA (9)	1.33 (0.76–2.32)	0.322
Top-quality embryos		
Others*	Reference	
4BB	1.38 (0.99–1.91)	0.059
4BA	**3.16 (1.47–6.77)**	**0.003**
4AB	1.19 (0.63–2.25)	0.599
4AA	1.13 (0.68–1.87)	0.630

Indicators with statistically significant differences are expressed in bold (*P* < 0.05).

* Others were embryos of quality lower than that of 4BB.

ICM: inner cell mass; TE: trophectoderm; OR: odds ratio; CI: confidence interval.

We also analysed the level of hCG on different days after frozen single blastocyst transfer and found that hCG levels at 14 days after ET were significantly different between the MZ twins and singleton pregnancy groups (Supplementary Table S1).

## Discussion

Advanced higher maternal age, lower paternal age, fresh ETs, blastocyst transfers, and higher quality blastocysts (4BA) all appeared to increase the chances of MZ twins.

The incidence of MZ twins after natural conception is about 0.4% (Hankins and Saade, 2005). However, our analyses showed an incidence rate of 1.638% (255 out of 15,567) for MZ twins after ART. This result was similar to the 1.53% reported in a study by Shi *et al.* (2021) (402 out of 26 254). In previous studies, however, the incidence of MZ twins after ART has been considerably higher. Liu *et al.* (2018) reported a rate of 2.69% (93 out of 3463). However, Ikemoto *et al.* (2018) showed that the incidence of multiple pregnancies with zygotic splitting was 1.36%. Thus, we can conclude that the incidence of MZ twins is much higher after ART compared to that with natural conception. The characteristics of the infertile population and procedures of ART may have an effect on the embryo division.

A considerable number of studies have aimed to understand the potential factors influencing the incidence of MZ twins. The division of embryos into MZ twins follows an autosomal dominant inheritance (Machin, 2009). Previous studies have found that MZ twins are not completely identical. The reasons may be differences in the intrauterine environment, genetic mutations and epigenetic differences. Furthermore, Liu *et al.* (2018) performed whole-genome sequencing of four consecutive generations from seven families and identified potential mutation loci on the X chromosome. These findings provide a new direction to explain some cases of MZ twinning. Sobek *et al.* (2015) suggested that hereditary factors were associated with the incidence of MZ twins and healthy ovarian function may further enhance the incidence. It has been reported that the incidence of MZ twins decreases by 4% with each increasing year of oocyte age (Knopman *et al.*, 2014). However, other studies have suggested that maternal age does not significantly affect the incidence of MZ twins (Nakasuji *et al.*, 2014; Mateizel *et al.*, 2016). In our study, advanced maternal age appeared to be associated with the risk of MZ twins. Conversely, paternal age appeared to be protective against MZ twins. A previous study has suggested that higher BMI and unexplained infertility may be risk factors for opposite-sex multiple pregnancies (Vega *et al.*, 2018). This was not seen in our analyses for same sex MZ twins.

A previous study reported that, with regard to gamete or embryo manipulation, the use of gonadotropins and the use of GnRH agonist or antagonist did not significantly change the incidence rate of MZ twins (Ikemoto *et al.,* 2018). Our results also suggested that different protocols of ovarian stimulation and the total hCG dose had no effect on the incidence of MZ twins. A study in Italy showed that ICSI was associated with a higher incidence of MZ twins when compared with IVF (Parazzini *et al.*, 2016). However, Busnelli *et al.* (2019) reported that compared with ICSI, the use of IVF was associated with a significantly increased incidence of MZ twins. The results of the present study showed that there were no significant differences in the incidence rate of MZ twins among different types of ART (IVF versus ICSI, and PGT), which was consistent with other previous studies (Mo *et al.*, 2015; Feng *et al.*, 2016; Ikemoto *et al.*, 2018).

The types of embryo transfer and culture time did influence the incidence rate of MZ twins. Using chi-squared analysis and binary logistic regression analysis, we found that FET may reduce the incidence of MZ twins. Consistently, Mateizel *et al.* (2016) found that the incidence of MZ twins after FET was approximately half of that after fresh ET. However, other studies have indicated that there are no statistical differences in the incidence rate of MZ twins between FETs and fresh ETs (Feng *et al.*, 2016). Our research showed that blastocyst transfers significantly increased the incidence of MZ twinning, which was consistent with most previous studies (Aston *et al.*, 2008; Hviid *et al.*, 2018; Busnelli *et al.*, 2019). The increased incidence may be because artificial culture conditions disrupt the communication between blastomeres and lead to the formation of two independent blastocysts. Some researchers believe that prolonged exposure to an artificial medium may result in an altered zona pellucida, which could increase the fragility and propensity of the embryo to separate (Aston *et al.*, 2008; Esfandiari *et al.*, 2009; Hviid *et al.*, 2018; Liu *et al.*, 2018; Busnelli *et al.*, 2019).

We also found that blastocyst grading had an effect on the incidence of MZ twins. The rate of monochorionic-diamniotic twins after transfer of a blastocyst with a high grade (A) of ICM has been reported to be 0.38%, which was significantly lower compared to that with poorer grades (B and C) of ICM (1.38%) (Otsuki *et al.*, 2016). We suggest that a C grade of TE may be protective against MZ twinning. Blastocysts with grades A and B for TE had a 1.6- to 1.9-fold higher chance of developing into MZ twins, respectively, compared to the TE grade C blastocysts. A similar trend was observed for ICM+TE and top-quality blastocysts. Blastocysts with better grades had a higher chance of developing into MZ twins. A recent study showed that the increased incidence of MZ twins is associated with a loosely arranged ICM and tightly packed TE cells. The authors proposed that higher quality embryos may produce more hCG, downregulate the level of intrauterine insulin-like growth factor binding protein-1, change endometrial receptivity and delay implantation time, which may explain why high grade of TE can increase the chance of MZ twinning (Shi *et al.*, 2021). We also suggest that the choice of high-quality blastocysts for transfer may be responsible for the higher incidence of MZ twins in pregnancies after ART than in natural pregnancies. This may be regarded as an adverse effect of human intervention.

The advantages of this study were that the sample size was relatively large, and we analysed the potential factors influencing incidence of MZ twins including characteristics of the infertile population, gamete and embryo manipulation and blastocyst scores. However, the study has limitations. Firstly, we defined MZ twins as twin births after single embryo transfer, rather than by an ultrasound examination in early pregnancy, which might be confounded by some cases of vanishing twin syndrome or the combination of a transferred embryo and sexual intercourse (Ikemoto *et al.*, 2018; Vega *et al.*, 2018; Zamani and Parekh, 2022). Secondly, the characteristics of the two groups were homogenous, and hence it was challenging to associate infertility factors with the incidence of MZ twins.

## Conclusions

Our analysis found that the incidence of MZ twins was higher after ART than that after natural conception. Advanced maternal age, fresh embryo transfer, blastocyst transfers, and high-quality embryos may be factors influencing the incidence of MZ twins. To avoid the complication of a twin pregnancy, choosing a single frozen embryo or single cleavage-stage embryo for transfer, particularly for people at high risk, may be beneficial to both the foetus and the mother.

## Supplementary Material

hoad035_Supplementary_Data

## Data Availability

The data underlying this article will be shared upon reasonable request to the corresponding author.
