# Supplementary material for: Risk factors associated with monozygotic twinning in offspring conceived by assisted reproductive technology
Source: Hum Reprod Open. 2023 Sep 14;2023(4):hoad035. doi: 10.1093/hropen/hoad035 (PMC10570986; doi:10.1093/hropen/hoad035)
Supplement: hoad035_Supplementary_Data [file hoad035_supplementary_data.docx]

|  | MZT  pregnancy | Singleton pregnancy | P-value |
| --- | --- | --- | --- |
| N | 199 | 877 |  |
| **hCG** (IU/L), mean±SD |  |  |  |
| D12 | 727.30±416.95 | 581.96±324.49 | 0.092 |
| D13 | 907.66±479.17 | 846.71±428.48 | 0.302 |
| **D14** | **1378.44±710.13** | **1101.04±725.82** | **0.011** |
| D15 | 1530.67±860.31 | 1512.25±973.06 | 0.694 |

**Supplementary Table S1. hCG levels on different days after frozen single blastocyst transfer**

Data are shown as mean ± SD. Indicators with statistically significant differences are in bold type (P < 0.05).

hCG: human chorionic gonadotropin

MZT: monozygotic twin
